# Human Omental Mesothelial Cells Impart an Immunomodulatory Landscape Impeding B- and T-Cell Activation

**DOI:** 10.3390/ijms23115924

**Published:** 2022-05-25

**Authors:** Benoit R. Gauthier, Diana Rubio-Contreras, Juan Carlos Gómez-Rosado, Luis Cristobal Capitán-Morales, Abdelkrim Hmadcha, Bernat Soria, Christian Claude Lachaud

**Affiliations:** 1 Andalusian Center for Molecular Biology and Regenerative Medicine-CABIMER, Junta de Andalucía-University of Pablo de Olavide-University of Seville-CSIC, 41092 Seville, Spain; 2 Biomedical Research Network on Diabetes and Related Metabolic Diseases (CIBERDEM), Institute of Health Carlos III, 28029 Madrid, Spain; khmadcha@upo.es (A.H.); bernat.soria@umh.es (B.S.); 3 Instituto de Biomedicina de Sevilla (IBiS), Hospital Virgen del Rocío-CSIC-Universidad de Sevilla, and Departamento de Genética, Universidad de Sevilla, 41013 Seville, Spain; dianarubio@us.es; 4 Departamento de Genética, Facultad de Biología, Universidad de Sevilla, 41012 Seville, Spain; 5 Unidad de Gestión Clínica de Cirugía General y Digestiva, Hospital Universitario Virgen Macarena, Avda. Dr Fedriani s/n, 41009 Sevilla, Spain; dr.gomez.rosado@gmail.com (J.C.G.-R.); lcapitan@us.es (L.C.C.-M.); 6 Departamento de Cirugía, Universidad de Sevilla, Avda. Dr Fedriani s/n, 41009 Sevilla, Spain; 7 General Hospital, Alicante Institute for Health and Biomedical Research (ISABIAL), 03010 Alicante, Spain; 8 Institute of Bioengineering and Health Research Institute (ISABIAL), Dr Balmis University Hospital (HGUA), Miguel Hernández University School of Medicine, 03010 Alicante, Spain

**Keywords:** mesothelial cells, adult stem cells, lymphocytes, macrophages, immunosuppression, immunomodulation

## Abstract

Mesothelial cells form the mesothelium, a simple epithelium lining the walls of serous cavities and the surface of visceral organs. Although mesothelial cells are phenotypically well characterized, their immunoregulatory properties remain largely unknown, with only two studies reporting their capacity to inhibit T cells through TGF-β and their consumption of L-arginine by arginase-1. Whether human mesothelial cells can suppress other immune cells and possess additional leukosuppressive mechanisms, remain to be addressed to better delineate their therapeutic potential for cell therapy. Herein, we generated secretomes from omental mesothelial cells (OMC) and assess their capacity to inhibit lymphocytes proliferation, suppress activated T and B cells, as well as to modify macrophage activation markers. The secretome from mesenchymal stromal cells (MSC) served as a control of immuno-suppression. Although OMC and MSC were phenotypically divergent, their cytokine secretion patterns as well as expression of inflammatory and immunomodulary genes were similar. As such, OMC- and MSC-derived secretomes (OMC-S and MSC-S) both polarized RAW 264.7 macrophages towards a M2-like anti-inflammatory phenotype and suppressed mouse and human lymphocytes proliferation. OMC-S displayed a strong ability to suppress mouse- and human-activated CD19^+^/CD25^+^ B cells as compared to MSC-S. The lymphosuppressive activity of the OMC-S could be significantly counteracted either by SB-431542, an inhibitor of TGFβ and activin signaling pathways, or with a monoclonal antibody against the TGFβ1, β2, and β3 isoforms. A strong blockade of the OMC-S-mediated lymphosuppressive activity was achieved using L-NMMA, a specific inhibitor of nitric oxide synthase (NOS). Taken together, our results suggest that OMC are potent immunomodulators.

## 1. Introduction

Mesothelial cells and their basement membrane form the mesothelium, a simple epithelium that lines the wall of serous cavities as well as visceral organs [1,2]. Although initially viewed as a support tissue, the mesothelium is now considered a highly dynamic tissue, whose functions are critical for the proper maintenance of body serous cavities [3,4,5]. A main function of mesothelial cells is to secrete large amounts of surfactant molecules, principally glycosaminoglycans that create a protective and viscous barrier that allows visceral organs to freely move inside serous cavities [2]. In addition to this mechano-structural function, several in vitro studies have shown that mesothelial cells can secrete either anti- or pro-inflammatory factors pending the environment [6,7,8,9]. In this context, co-injection of Freund adjuvant, a potent immunopotentiator, in combination with GM-CSF inhibits expression of IL-10 in rat peritoneal mesothelial cells while gaining strong IL-6 expression [8]. Additionally, it was demonstrated that peritoneal mesothelial cells secrete a relevant quantity of colony-stimulating factor 1 (CSF1) which strongly induces macrophages’ proliferation and regulates their homeostatic maintenance [10]. Despite these advances, little is known on the biological role of the different cytokines or inflammatory mediators secreted by mesothelial cells in either regulating the homeostasis of serous cavities or the activation state of serous fluid immune cells.

In recent years, the development of novel mesothelial-cell-based therapies for the treatment of human diseases has come into the limelight. Indeed, mesothelial cells fulfill diverse criteria for cell types considered with “high therapeutic potential” in regenerative medicine, as in the case for mesenchymal stromal cells (MSC). A key advantage of mesothelial cells is accessibility from diverse sources and ease of expansion in vitro [11,12,13]. In this context, the greater omentum is a large peritoneal fold containing abundant adipose tissue and is considered an optimal source from which omental mesothelial cells (OMC) can be harvested in therapeutically relevant numbers and with minimal health concerns [11,12,14]. Mesothelial cells possess significant plasticity as indicated by their capacity to differentiate in vitro and acquire features of vascular smooth muscle cells, osteocytes, adipocytes, and chondrocytes [13,15,16,17]. Of particular interest, mesothelial cell transplantation in a rat myocardial infarction model was shown to promote local tissue regeneration [18,19]. Several studies have also demonstrated that autologous peritoneal grafts efficiently prevented post-operative peritoneal adhesions [14,20,21]. As such, current developments in mesothelial-cell-based therapies are principally focusing on tissue engineering of autologous mesothelial cell sheets for the regeneration of the damaged mesothelium [5,22,23]. Notwithstanding, as the mesothelium also share morpho-structural and biochemical similarities with other simple epitheliums, mesothelial cells have also been highlighted as useful surrogate cells to regenerate the corneal endothelium, vascular endothelium, or synovium [24,25,26,27].

Although mesothelial cells have been well defined phenotypically and are already engaged in the development of diverse therapeutic applications, very little is known about their immunoregulatory properties and whether they could be useful cells for the treatment of autoimmune disorders. In support of such function, OMC can efficiently suppress reactive T-cell responses through action of TGF-β1 while human mesothelial-like cells derived from peritoneal fluid cells can efficiently suppress the proliferation of CD3-activated peripheral blood lymphocytes [28,29]. The lymphosuppressive activity of the mesothelial-like cells was attributed to high expression of arginase-1, which leads to L-arginine deprivation from the culture media, an amino acid essential for the survival and proliferation of lymphocytes [30,31]. More recently, mesothelial cells were shown to regulate the immune peritoneal homeostasis through secretion of diverse immune regulators such as IL-6, CSF1 further substantiating their immunoregulatory properties [9,10].

In order to further grasp the immunomodulatory function conveyed by human mesothelial cells, herein we focused on the capacity of the secretome released by human OMC to inhibit the activation and proliferation of lymphocytes, as well as to modify the phenotype of macrophages. The latter was compared and contrasted to the MSC-derived secretome, a reference for immunosuppression [32]. Despite the fact that OMC and MSC are defined as two distinct mesodermal cell types, we find that their Th1/Th2/Th17 cytokine secretory profiles are similar. Accordingly, the OMC- and MSC-derived secretomes were equally efficient in inhibiting the proliferation of both activated mouse and human lymphocytes. Nonetheless, the OMC-derived secretome suppressed mouse activated B cells more efficiently than the MSC-derived secretome. We also observed that the lymphosuppressive activity of OMC-derived secretome could be counteracted through inhibition of TGFβ or by the addition of L-arginine [28,29]. Furthermore, we found that the immunosuppressive capacity of OMC-derived secretome was efficiently ablated by blocking endogenous nitric oxide synthases (NOS) in lymphocytes. Taken together, our results suggest that OMC are potent immunomodulators.

## 2. Results

### 2.1. Omental-Derived Mesothelial Cells Exhibit a Typical Phenotype

**Figure 1:**
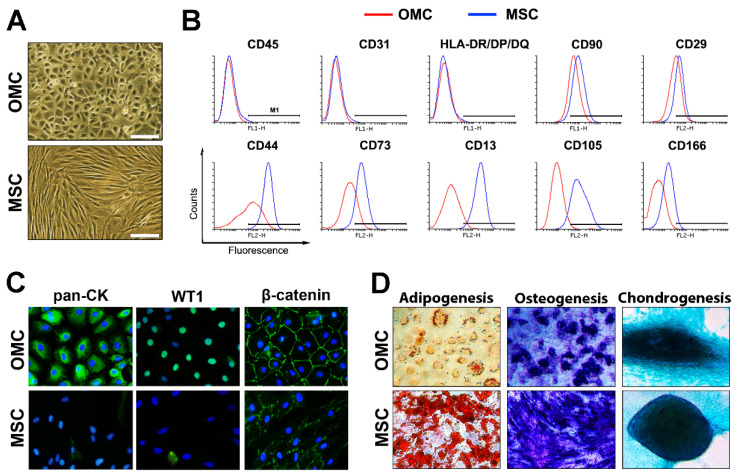
Phenotypic characterization of human omental mesothelial cells (OMC) and human mesenchymal stem cells (MSC). (**A**) Phase contrast pictures of confluent OMC and MSC cultures displaying typical cobblestone-type and fibroblastic morphologies, respectively. Scale bar is 100 µm. (**B**) Flow cytometric analysis of OMC and MSC for expression of hematopoietic (CD45), endothelial (CD31), HLA class II (HLA-DR/DP/DQ), and stromal/mesenchymal (CD90, CD29, CD44, CD73, CD73, CD13, CD105, and CD166) cell markers. Histograms for OMC and MSC are red and blue, respectively. M1 bar marks positivity delimited from isotype histograms (not shown). (**C**) Immunofluorescence analysis of OMC and MSC showing highly expressed epithelial (pan-cytokeratin or pan-CK) and mesothelial (Wilm’s tumor protein 1 or WT1) cell markers in OMC, but not in MSC, while the cell–cell junction protein β-catenin was expressed in both cell types. (**D**) Multilineage differentiation assay of OMC and MSC revealed limited adipogenic differentiation of OMC (Oilred O staining) compared to MSC. In contrast, OMC displayed more similar osteogenic and chondrogenic differentiation compared to MSC. (**A**–**C**) OMC used are ALIC1 cells.

Human mesothelial cells were isolated from the greater omentum of several donors and expanded in culture. Similar to mesothelial cells derived from the pleural cavity [33], OMC typically formed a cobblestone-like monolayer at confluency with no evidence of fibroblast contamination. By contrast, human adipose-tissue-derived MSC displayed a fibroblastic shape (Figure 1A). We next phenotypically characterized and compared to MSC these newly established cell lines, including their capacity to differentiate into several cell lineages. Both OMC and MSC lacked expression of the cell surface markers, HLA-DR/DP/DQ (MHC-II receptors) CD45 (hematopoietic) and CD31 (endothelial) whereas they expressed similar levels of the stromal cell markers CD90 and CD29 (Figure 1B). Consistent with their developmental origin, MSC expressed higher levels of the stroma/mesenchymal markers CD44, CD73, CD13, and CD105 as compared to OMC (Figure 1B). In contrast, cytokeratin (epithelial) and WT1 (mesothelial) were only expressed in OMC (Figure 1C). Interestingly, beta-catenin was highly expressed at the cell surface of OMC while only low levels could be discerned on MSC (Figure 1C). In contrast to previous reports using pericardial-fluid-derived mesothelial cells [16], OMC poorly differentiated into adipocytes as compared to MSC while readily acquired either an osteocyte or chondrocyte lineage (Figure 1D). Taken together, these results establish the bona fide mesothelial phenotype of OMC with stem/progenitor characteristics and differentiation potential.

### 2.2. Omental Mesothelial Cells Display a Mixed Pro- and Anti-Inflammatory Signature

**Figure 2:**
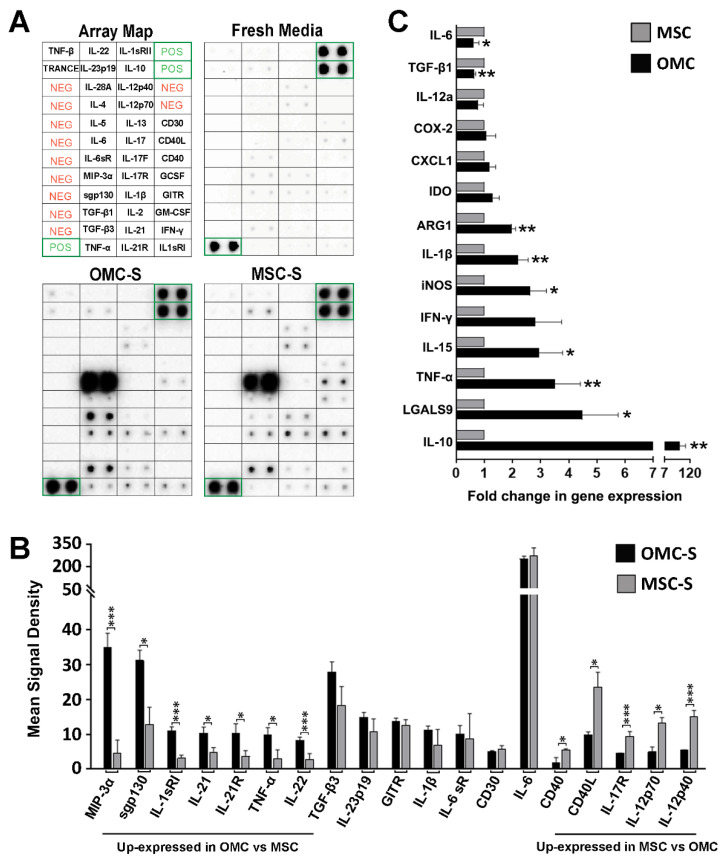
OMC and MSC display similarities in their expression patterns of anti- and pro-inflammatory markers. (**A**) Representative membrane antibody arrays incubated within fresh media, OMC- or MSC-derived secretome (OMC-S and MSC-S, respectively). Array map (upper left) is shown for cytokine localization and positive (POS) and negative (NEG) controls. (**B**) Quantification of cytokines expressed in OMC-S and MSC-S. Results are shown as mean ± s.e.m of signal densities from (*n* = 3) independent batches of ALIC1 OMC-S and MSC-S. (**C**) Quantitative PCR analysis of the expression of pro- and anti-inflammatory and immunoregulation genes in OMC and MSC. Results are shown as mean fold change ± s.e.m in mRNA expression relative to MSC (values set as 1), from (*n* = 4) distinct batches of ALIC1 OMC and MSC cultures. (**B**,**C**) Statistical significance was determined by Student’s *t*-test. * is for *p* ≤ 0.05; ** is for *p* ≤ 0.01; *** is for *p* ≤ 0.001.

In order to better grasp the immunomodulatory function potentially conveyed by OMC, we profiled the Th1/Th2/Th17 cytokines secreted by OMC and compared it to that of MSC (Figure 2A,B).

Although globally the cytokine secretion profiles were similar, some important differences were discerned between OMC- and MSC-derived secretomes (OMC-S and MSC-S, respectively) (Figure 2A,B). As such, OMC-S and MSC-S secreted similarly high levels of interleukin-6 (IL-6), a pleiotropic cytokine with context-dependent pro- and anti-inflammatory properties [34]. Both cell types also secreted similarly high levels of TGFβ3 (Figure 2B). In contrast, secretion of MIP-3α (CCL20), Sgp130, IL-1sRI, TNF-α, IL-21, and IL-21R was significantly higher in OMC as compared to MSC whereas CD40, CD40L, IL-17R, IL-12p70, and IL-12p40 secretion was higher in MSC (Figure 2B). We next assessed transcript levels of several key inflammatory and immunomodulary genes (Figure 2C). OMC expressed significantly higher levels of *IL-15* (T cells activation), *IL-1β*, *IFNγ*, and *TNFα* (pro-inflammatory cytokines), iNOS (inflammatory and immunosuppression), and *ARG1*, *LGALS9*, and *IL-10* (anti-inflammatory). Of particular interest, the expression of the potent anti-inflammatory IL-10 cytokine was 100-fold higher in OMC as compared to MSC.

### 2.3. The Secretome of OMC Induces M2 Polarization and Inhibits Mouse Lymphocyte Proliferation and Agglutination

**Figure 3:**
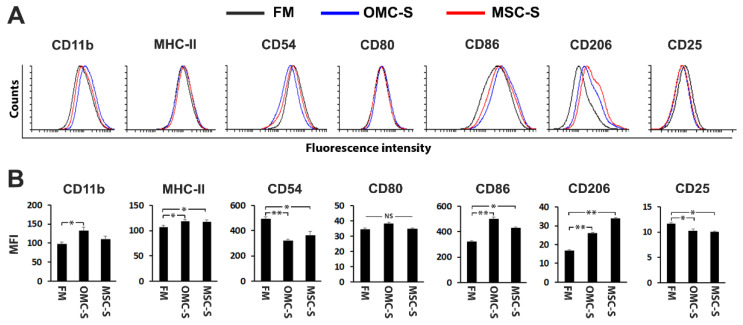
OMC-derived secretome favors an anti-inflammatory M2 phenotype. (**A**,**B**) RAW264.7 cells were cultured for 6 days in fresh media (FM) and either OMC- or MSC-derived secretome (OMC-S and MSC-S, respectively). Expression levels of CD11b, MHC-II, CD54 (M1 associated marker), CD80, CD86, CD206 (M2 associated marker), and CD25 were assessed by flow cytometry. (**A**) Representative histograms and (**B**) quantification of mean fluorescence intensity (MFI) are depicted. Isotype histograms are not shown. Results are shown as mean ± s.e.m of (*n* = 3) independent batches of OMC-S (from ALIC1, SEV1, and SEV3 OMC lines) and MSC-S production. Statistical differences between conditions were calculated using Student’s *t* test; * is for *p* ≤ 0.05; ** is for *p* ≤ 0.01; NS, not significant.

In order to determine whether the OMC secretome conveys either pro- or anti-inflammatory properties, mouse Raw 264.7 cells were exposed to condition media obtained from OMC (OMC-S) cultures. Raw 264.7 cells cultured in the presence of OMC-S displayed reduced expression of the M1 pro-inflammatory markers CD54 and of the activator marker CD25 [35]. In contrast, expression of the M2 anti-inflammatory marker CD206 was increased as compared to cells that we cultured in fresh media (FM) (Figure 3A,B).

MSC-S induced similar changes in the expression pattern of these markers (Figure 3A,B). Interestingly, markers of macrophage activation, MHC-II and CD86, but not CD80 were also upregulated by both OMC-S and MSC-S (Figure 3A,B).

**Figure 4:**
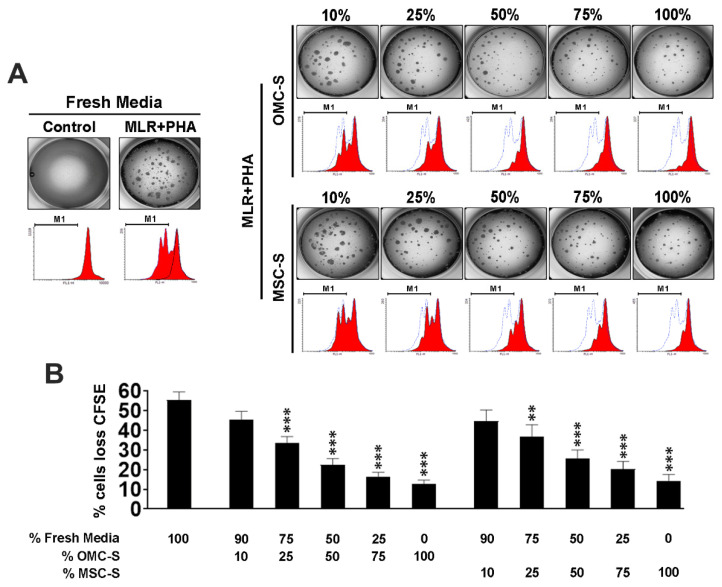
The OMC-derived secretome inhibits mouse lymphocytes proliferation. (**A**) Left upper panel depicts representative images of lymph nodes (LN) lymphocytes cultured in the absence or presence of 25 µg/mL phytohemagglutinin (PHA) and MLR. Lower images depict histograms of carboxyfluorescein succinimidyl ester (CFSE) expression after 72 h of culture, showing loss of CFSE expression in the MLR + PHA condition (dashed black line shows LN lymphocytes just after initial CFSE labeling). Right panel, upper images, show images of MLR + PHA cultures performed with increasing amounts of OMC- or MSC-derived secretome (OMC-S or MSC-S, respectively). Lower images, corresponding CFSE histogram expression. Dashed blue line is for CFSE expression level of the MLR + PHA control condition in 100% fresh media. (**B**) Summary quantification of CFSE^low^ proliferating lymphocytes in MLR + PHA cultures performed in 100% fresh media or increased proportions of OMC-S or MSC-S. Results shown are mean ± s.e.m percentages of cells with CFSE loss (CFSE^low^) after 72 h of culture, from (*n* = 3) independent OMC-S batches from ALIC1 and SEV1 OMC lines. Statistical differences against control (100% fresh LM) were calculated with ANOVA. ** is for *p* ≤ 0.01; *** is for *p* ≤ 0.001.

Overall, the OMC immunomodulatory secretory profile appears to convey an anti-inflammatory M2 phenotype to Raw 264.7 cells. To further substantiate this premise, we assessed the capacity of OMC to inhibit lymphocyte proliferation. To this end, lymphocytes isolated from lymph nodes of C57BL6 and FVB mice were fluorescently labeled using CFSE and subjected to a two-way mixed lymphocyte reaction (MLR) culture containing phytohemagglutinin (PHA) to induce their agglutination and promote a strong proliferation induced by double polyclonal activation (Figure 4A). Remarkably, OMC-S produced a clear dose-dependent reduction in lymphocytes agglutination and the percentages of CFSE^low^ lymphocytes, indicative of blunted proliferation (Figure 4B,C). Similar effects were observed with MSC-S (Figure 4B,C). Consistent with their reduced agglutination, further experiments indicated that the expression of CD54/ICAM-1 which is associated with cell–cell adhesion, was robustly inhibited by OMC-S (Figure S1). Taken together, these results suggest that OMC convey anti-, rather than pro-, inflammatory properties.

### 2.4. Omental Mesothelial Cell Secretome Inhibits the Activation of Mouse T and B Cells

**Figure 5:**
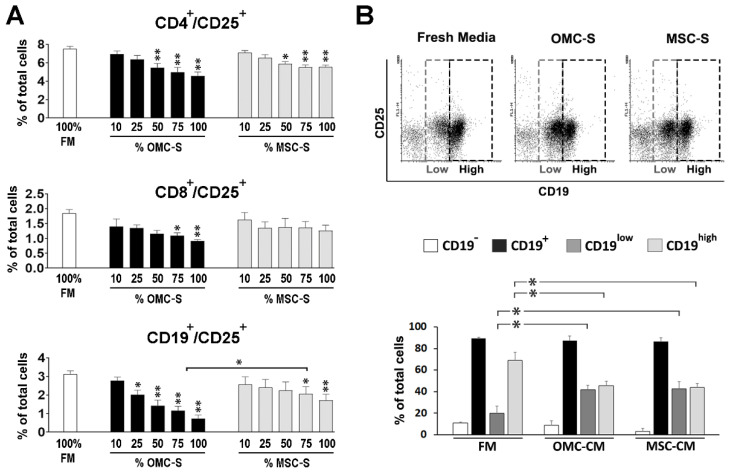
OMC-derived secretome inhibits the activation of lymph node T and B cells and peritoneal fluid B cells. (**A**) Quantification (% of total cells) of CD4^+^/CD25^+^ and CD8^+^/CD25^+^ activated T cells and CD19^+^/CD25^+^ activated B cells within mouse lymph nodes (LN) cells cultured for 24 h in 100% fresh media supplemented with 25 µg/mL phytohemagglutinin (PHA) or increased proportions (10–100%) of OMC-S (ALIC1, SEV1, and SEV4) or MSC-S supplemented with PHA. Results are mean ± s.e.m from *n* = 4 independent experiments. Statistical differences between control (100% fresh media + PHA) and OMC-S and MSC-S conditions were calculated using ANOVA. Difference between 100% OMC-S and MSC-S for CD19^+^/CD25^+^ B cells was calculated with Student’s *t* test. (**B**) OMC-derived secretome inhibits peritoneal fluid B cells. Upper, shows representative coexpression of CD19 and CD25 in non-adherent mouse peritoneal fluid cells (PFC) cultured for 72 h in either fresh media (FM), OMC-S, or MSC-S. Two rectangles delimiting CD19^low^ (grey rectangle) and CD19^high^ (dark rectangle) expressing B cells are shown. Lower graph shows flow cytometric quantification of CD19^−^, CD19^+^, CD19^low^, and CD19^high^ populations within non-adherent PFC cultured for 72 h in either fresh media or OMC-S (ALIC1, SEV1, and SEV4) and MSC-S. Results are shown as mean ± s.e.m from *n* = 3 independent experiments. Statistical differences for CD19^low^ and CD19^high^ between different culture conditions groups were calculated using Student’s *t* test. (**A**,**B**) * for *p* ≤ 0.05; ** for *p* ≤ 0.01.

To further delineate the lymphocyte subpopulations targeted by OMC-S, we analyzed the ability of OMC-S to inhibit T- and B-cell activation in mixed T and B lymph node cell populations activated with PHA. Interestingly, the reduction of PHA-induced agglutination of lymphocytes provoked by OMC-S was also accompanied by a significant reduction of CD4^+^/CD25^+^ and CD8^+^/CD25^+^ activated T cells as well as CD19^+^/CD25^+^ B cells (Figure 5A). While activation of CD4^+^ and CD8^+^ T cells was as equally reduced by MSC-S as OMC-S, inhibition of CD19^+^ B cell activation was significantly higher in 100% OMC-S as compared to 100% MSC-S (Figure 5A). In further support of these results, OMC-S was also found to significantly inhibit mouse peritoneal fluid CD19^+^ B cells, as evidenced by their significant increase in B cells displaying loss of CD19 expression (Figure 5B).

### 2.5. Activation and Proliferation of Human Lymphocytes and B Cells Are Blunted by OMC-S

**Figure 6:**
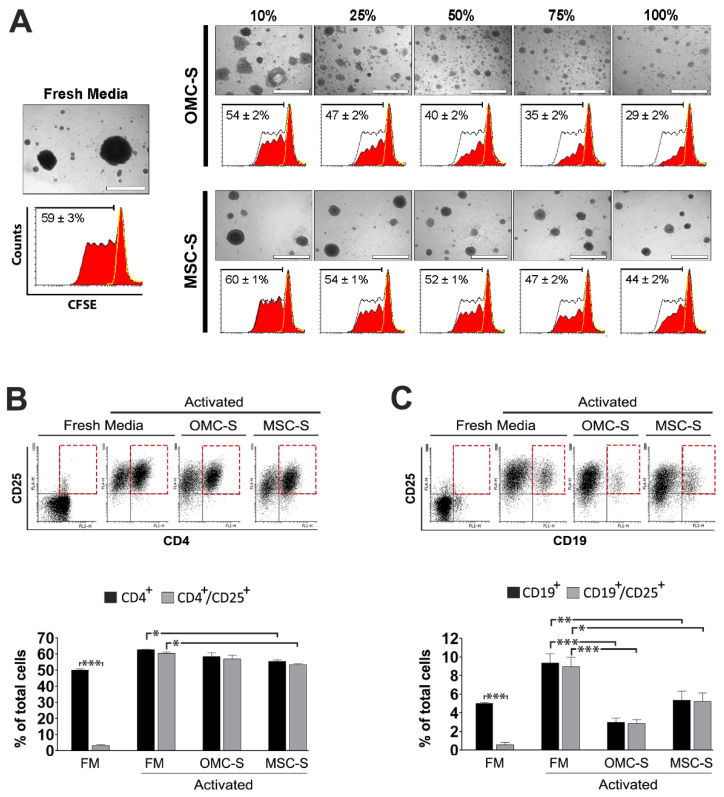
OMC-derived secretome inhibits the proliferation of human lymphocytes and the activation of B cells. (**A**) Representative images (upper panel) of a mixed lymphocyte reaction (MLR) of PBMC-enriched lymphocytes labeled with CFSE and cultured in either 100% fresh media (FM) supplemented with 25 µg/mL phytohemagglutinin (PHA) or increased amounts of OMC-S and MSC-S supplemented with PHA. Scale bar is 500 µm. Lower histograms depict corresponding CFSE expression levels (red histograms) of disaggregated lymphocyte cultures. The yellow dashed line marks cells freshly labeled with CFSE. For reference, a black dashed line corresponding to CFSE expression histogram in 100% FM is shown in OMC-S and MSC-S histograms. Results are shown as mean percentages ± s.e.m of CFSE^low^ cells calculated from *n* = 2 independent batches of OMC-S (ALIC1, SEV1) and MSC-S. (**B**,**C**) OMC-S efficiently suppress B cells activation. Human PBMC-enriched lymphocytes subjected to double polyclonal stimulation (anti-CD3/CD28; PHA) were expanded for 6 days and finally incubated for 2 days in FM, OMC-S, or MSC-S. Cells were analyzed by flow cytometry. (**B**) Analysis of CD4 and CD25 coexpression. Upper panel shows representative dot plots of CD4/CD25 expression. Lower graph, quantification of mean percentages of total CD4^+^ T cells and CD4^+^/CD25^+^-activated T cells. (**C**) Flow cytometry analysis of CD19 and CD25 coexpression. Upper panel shows representative dot plots of CD19/CD25 expression. Lower graph, quantification of mean percentages of total CD19^+^ B cells and CD19^+^/CD25^+^ activated B cells. (**B**,**C**) Results are mean ± s.e.m results of *n* = 3 distinct batches of OMC-S (ALIC1, SEV1, and SEV3) and MSC-S. Statistical differences were calculated using ANOVA. * is for *p* ≤ 0.05; ** for *p* ≤ 0.01; *** *p* ≤ 0.01.

We next sought to translate our murine data to human immune cells. To this end, peripheral blood mononuclear cells (PBMC)-enriched lymphocytes from two distinct donors were subjected to a two-way mixed lymphocyte reaction (MLR) culture containing PHA to robustly induce proliferation (Figure 6A).

Consistent with our mouse data, both the PHA-induced agglutination and proliferation of human lymphocytes were dose-dependently reduced by OMC-S (Figure 6A). We also determined whether OMC-S could lower the activation of PBMNC-enriched lymphocytes after CD3/CD28-mediated polyclonal activation and PHA stimulation. As expected, polyclonally (TCR and PHA) activated lymphocytes cultured in fresh media (FM) proliferated and generated large free-floating clusters composed mainly of CD3^+^ and CD4^+^ T cells and a minor CD19^+^ B cells subpopulation (Figure S2). The vast majority of CD4^+^ T cells within FM/TCR/PHA cultures were also CD25^+^ (Figure 6B). Although not significant, OMC-S impeded expansion of the CD4^+^/CD25^+^ T cells subpopulation while MSC-S significantly blunted its proliferation (Figure 6B). In contrast, activation of the CD19^+^/CD25^+^ B cell subpopulation was significantly reduced by OMC-S and to a lesser extent by MSC-S (Figure 6C).

### 2.6. The Lymphosuppressive Activity of OMC-Derived Secretome Is Conveyed via TGFβ as Well as Nitric Oxide Synthase Activity

**Figure 7:**
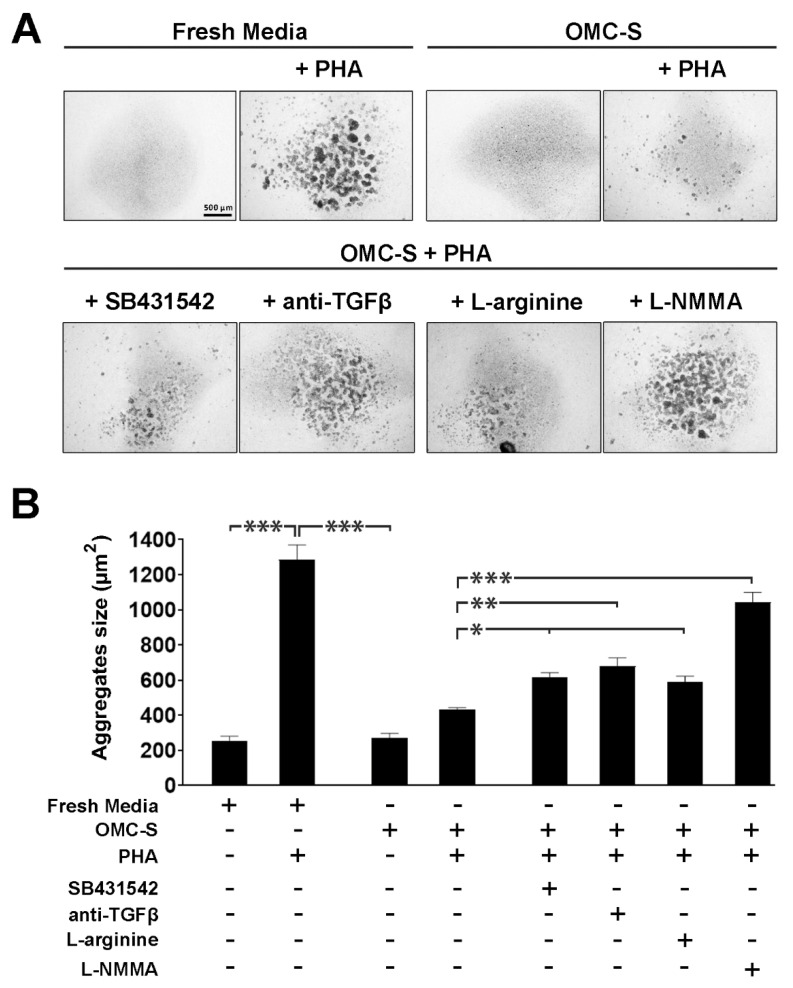
Lymphosuppression of OMC-derived secretome is partially mediated through TGFβ, L-arginine, and induction of nitric oxide synthase (NOS) in lymphocytes. (**A**). Upper panels, low-magnification (2×) images of mouse lymph nodes (LN) lymphocytes cultured for 48 h in fresh media or OMC-derived secretome (OMC-S), with or without 25 µg/mL phytohemagglutinin (PHA). Scale bar: 500 µm. Lower panel, images of mouse LN lymphocytes cultured in the presence of OMC-S + PHA supplemented with 50 µM SB-431542 (selective inhibitor of ALK5/TGF-β1 Receptor), 1 µg/mL of a mouse monoclonal anti-TGF- β1, β2, and β3 antibody, 1 mM L-arginine, or 100 µM L-NMMA (specific inhibitor of nitric oxide synthase). (**B**) Quantification of lymphocyte agglutination in response to the various experimental culture conditions by measuring mean particle size surface (µm^2^). Results shown are mean ± s.e.m from (*n* = 3) distinct batches of OMC-S from ALIC1, SEV3, and SEV4. Statistical differences between experimental conditions were calculated using one way ANOVA. * is for *p* ≤ 0.05; ** for *p* ≤ 0.01; *** *p* ≤ 0.001.

We next sought to identify potential molecular pathways mediating the lymphosuppressive activity of OMC-S (Figure 7). We initially focused on the TGF-β signaling cascade as TFG-β3 secretion was elevated in both OMC-S and MSC-S (Figure 2B). Inhibition of TGF-β via the use of either the selective inhibitor, SB-431542, or a monoclonal anti-TGF-β antibody, blunted the immunosuppressive activity of OMC-S as assessed by reduced agglutinated particle sizes (Figure 7A,B). We next assessed the effect of arginine supplementation on OMC-S-mediated inhibition of cell aggregation. Indeed, *ARG1*, a key gene involved in inflammation resolution and for which the encoded enzyme converts arginine to ornithine thereby depleting arginine pools, was significantly increased in OMC as compared to MSC (Figure 2C). Accordingly, addition of L-arginine blunted the OMC-S-mediated inhibition of cell agglutination (Figure 7A,B). Interestingly, addition of L-NMMA, a specific inhibitor of the nitric oxide synthase (NOS), strongly abrogated the immunosuppressive effect of OMC-S (Figure 7A,B). Taken together, these results indicate that OMC-S exerts its lymphosuppressive activity through several independent signaling pathways, including the likely overstimulation of NOS activity in lymphocytes, leading to supraphysiological toxic nitric oxide levels that limit cell activity.

## 3. Discussion

Herein, we characterized human OMC and studied their immunosuppressive capacity. Human mesodermal-derived MSC were used as reference for comparison [32,36]. Consistent with different mesodermal origins, OMC and MSC displayed distinct morphologies as well as expression levels for several mesenchymal, stromal, epithelial, and mesothelial cells markers. For example, we found that several stromal and/or mesenchymal markers (CD44, CD73, CD13, CD105, and CD166) were expressed at much lower levels by OMC as compared to MSC and could therefore prove to be useful cell-surface markers to discriminate OMC from MSC. In addition, OMC displayed decreased adipogenesis potential, further substantiating differences with MSC.

Despite their phenotypic differences, OMC and MSC displayed many similarities in their cytokine secretion profiles. As such, both cell-derived secretomes contained very high levels of interleukin-6 (IL-6), a pleiotropic cytokine shown to stimulate B cell activation and growth [34,37]. In contrast to this reported IL-6 mediated-activation of B cells, the OMC-derived secretome significantly inhibited both human and mouse CD19^+^/CD25^+^ activated B cells suggesting that additional factors secreted by OMC supersede the effect of IL-6. Consistent with this premise, the OMC-, as opposed to the MSC-derived secretome contained high levels of soluble gp130 (sgp130) which was shown to inhibit IL-6 actions [38] suggesting that the overall effect of the OMC secretome is to establish an anti-inflammatory environment. Notwithstanding, B-cell activation observed in either PHA or anti-CD3/28 activation experiments could likely stem from an indirect effect arising from T-cell activation in the culture. Further studies involving direct B-cell stimulation using either anti-IGM or IL-4, for example, are needed to explore the direct suppressive effect of OMC-S on this cell type. Independently of activation stimuli, our findings are particularly relevant as B cells, and more particularly B1a lymphocytes, are abundant in the serous fluid of the peritoneal and pleural cavities [39,40], which foster the prospect that mesothelial cells could be an important checkpoint to harness the aberrant activation of B in the omentum environment prone to mechanical inflammation.

In line with the anti-inflammatory properties of OMC, high levels of TGFβ were also secreted by these cells. This growth factor was shown to polarize macrophages towards an M2 phenotype [41]. Accordingly, OMC-derived secretome polarized RAW264.7 towards the M2 phenotype, as assessed by the upregulation of the M2 marker CD206 and downregulation of the M1 marker CD54 [42]. In addition, TGFβ depletion using either an inhibitor or antibody resulted in the partial loss of the secretome-mediated inhibitory action on lymphocyte proliferation substantiating a previous study [28]. As such TGFβ, along with sgp130, is an important factor contributing to the overall anti-inflammatory landscape imparted by OMC. In this context, high levels of MIP-3α macrophage inflammatory protein 3 alpha, also known as CCL20, a chemoattractant for dendritic, B and T cells as well as macrophages were secreted to a higher extent by OMC as compared to MSC. This chemokine acts as an anti-viral and -microbial factor at mucosal surfaces [43]. As such, MIP-3α within the OMC secretome may participate in the recruitment of lymphocytes and B cells, which are maintained in an anti-inflammatory state by other secreted factors such as TGFβ and sgp130 while also safeguarding the omentum from bacterial and viral invasion.

We also found that the supplementation of the OMC-derived secretome with L-NMMA, a specific inhibitor of the nitric oxide synthase, strongly reduced its lymphosuppressive activity. Since L-NMMA directly acts on lymphocytes, these results suggest that the OMC-derived secretome inhibits lymphocytes through induction of their endogenous NOS activity, thereby generating deleterious high intracellular levels of nitric oxide in lymphocytes, which finally induce their inhibition. Nitric oxide has well-known immunomodulatory activity on T cells, leading to their inhibition at strong concentration [44]. It has already been reported that the overproduction of nitric oxide (NO) mediates the collapse of lymphoproliferative responses in a rat model of thermal injury [45,46]. The authors showed that high endogenous NO production led to the arrest of activated T cells in the G1 phase of the cell cycle and prompted apoptosis. The use of NO synthase inhibitors could antagonize alterations of cell proliferation and cell death parameters in burned rat T cells. Inducible nitric oxide synthase (iNOS) is widely expressed in immune cells and regulated at the transcriptional level by different stimuli, including pro-inflammatory cytokines such as IFN-γ, IL-1β, and TNF-α [47]. IFN-γ and TNF-α were also shown to strongly induce endogenous nitric oxide synthase in macrophages or dendritic cells, leading to their inhibition and apoptosis [48,49]. Accordingly, high levels of *TNF-α* transcript as well as secreted proteins were detected in OMC as compared to MSC. As such, it is therefore possible that such mechanism could also be used by OMC to exert their immunosuppressive activity. Further studies addressing this mechanism may bring light on whether peritoneal mesothelial cells use TNF-α secretion to suppress peritoneal lymphocytes through their NOS activity.

Overall, our study shows that the secretome of omental mesothelial cells (OMC) cultured under standard conditions possess significant immunoregulatory activities that inhibit pro-inflammatory macrophages, T cells, and B cells.

## 4. Materials and Methods

### 4.1. Isolation of Human Omental Mesothelial Cells (OMC)

Human omental mesothelial cells (OMC) were isolated from biopsies (10–20 g) of the greater omentum. A first OMC line was derived from omental tissue donated from a young woman (25 years old) after cesarean delivery, from Alicante, Spain, and was termed ALIC1. Following, OMC lines (SEV1-6) were derived from omental tissue donated by 6 adult patients (60 ± 4 years old) undergoing surgery for non-tumoral peritoneal disorders at the unit of Colorectal Surgery Unit, Virgen Macarena University Hospital, Seville, Spain. All patients were informed and gave their written consent. A total of 4 OMC primary cell lines with optimal parameters were used in this study (ALIC1, SEV1, SEV3, and SEV4). Optimal parameters for characterizing OMC lines were uniform epithelial morphologies with cobblestone-like morphology at confluence and wide and strong expression of pan-cytokeratin and β-catenin. Three OMC lines (SEV1, SEV3, and SEV4) derived from the Colorectal Surgery Unit were deposited at the Andalusian Biobank, Seville, Spain. Omental tissue biopsies were collected into DMEM GlutaMax^TM^ low glucose media (Gibco, 21885) with 1% penicillin streptomycin (P/S) and stored on ice during shipment to the culture room. Upon reception, the omental tissue was washed in phosphate-buffered saline solution (PBS) and incubated for 20 min into a 50 mL conical tube containing 30 mL of a 37 °C pre-heated enzymatic solution (PBS calcium free +2% bovine serum albumin (BSA) +0.25% trypsin). Optimal OMC detachment was achieved through gentle shaking of the tube each 5 min. Detached OMC were recovered after collection and centrifugation of the enzymatic solution (500× *g*; 5 min). Viable cells were counted with the trypan blue exclusion assay.

### 4.2. Culture of Human OMC

Freshly isolated cells were seeded at high density (50,000 cells/cm^2^) in a modified mesothelial growth media (MGM) formulated to stimulate OMC proliferation while preventing their epithelial-to-mesenchymal transition [50]. MGM is a DMEM, low glucose, GlutaMAX^TM^ (21885, Gibco, Loughborough, UK) supplemented with 10% heat inactivated fetal bovine serum or FBS (Hyclone); 1× Penicillin–Streptomycin (P/S; Gibco); 50 μM beta-mercaptoethanol (Gibco); 1× ITS-A (Gibco; 51300); 10 ng/mL of human recombinant EGF (AF-100-15, PeproTech, London, UK) and 0.1 μg/mL hydrocortisone (H0888, Sigma-Aldrich, St. Louis, MO, USA). Confluent OMC were then subcultured (seeding at 20,000 cells/cm^2^) in MGM until subculture passage 3 (P3), being at that step highly homogeneous and stabilized in the typical cobblestone morphologies. OMC were subjected to an additional subculture (P4) in MGM without hydrocortisone, after when OMC were harvested and used for conditioned media production.

### 4.3. Culture of Human Mesenchymal Stem Cells (MSC)

Primary human mesenchymal stem cells (MSC) derived from subcutaneous adipose tissue acquired by liposuction (ATCC^®^ PCS-500-011) were used as a reference of cells with immunosuppressive properties [32,36]. MSC at subculture passage 4 (P4) were used for conditioned media production. MSC expansion culture media used was a DMEM, low glucose, GlutaMAX^TM^ supplemented with 10% FBS, 1× P/S, 50 μM beta-mercaptoethanol, and 1× ITS-A. Subconfluent MSC were detached with 0.05% trypsin and subcultured at seeding density of 10,000 cells/cm^2^.

### 4.4. Production of OMC- and MSC-Derived Secretomes

OMC- and MSC-derived conditioned media or secretomes (OMC-S and MSC-S, respectively), were produced in independent batches (*n =* 14). To this end, 1.4 × 10^6^ OMC and MSC were resuspended in 5 mL of fresh media (FM) and plated into 6 cm diameter Petri dishes (seeding density of 50.000 cells/cm^2^). The FM formulation was DMEM, low glucose, GlutaMAX^TM^ basal media supplemented with 10% FBS; 50 µm 2-mercaptoethanol; 1× HEPES; 1× sodium pyruvate and 1X P/S (all from Gibco). OMC-S and MSC-S were collected at 24 h, centrifuged (300× *g*; 5 min), and supernatant aliquots stored at −80 °C. The same volume of fresh media was added to cells and conditioned media collection repeated similarly after 24 h and stored at −80 °C. A total of 12 independent batches of OMC-S from distinct OMC primary lines and MSC-S (PCS-500-011 MSC) were produced. For immunosuppression experiments, aliquots of OMC-S or MSC-S collected at 24 and 48 h from the same batch were thawed and mixed just before experimental use. OMC and MSC used for conditioned media production were finally harvested at 48 h with 0.05% trypsin and characterized by flow cytometry and qPCR.

### 4.5. Immunofluorescence Characterization of OMC and MSC

Immunofluorescence characterization of OMC and MSC was performed using subcultured P4 cells grown into hydrophilic µ-Dish (45079, Ibidi GmbH, Gräfelfing, Germany). For intracellular antigens detection, cells were fixed with 4% paraformaldehyde (PFA) and permeabilized with 0.5% Triton X-100 (Sigma-Aldrich Corp.) or fixed and permeabilized with cold methanol (−20 °C). Cells were then blocked in PBS-BSA prior to incubation with primary and secondary antibodies (Supplementary Table S1). Nuclei were counterstained with 1 μg/mL Hoechst 33,342 (Sigma-Aldrich). Fluorescence images were captured with an Olympus IX71 inverted fluorescence microscope (Olympus, Tokyo, Japan).

### 4.6. Flow Cytometry

OMC and MSC at subculture passage 4, as well as human or mouse T and B cells were characterized by flow cytometry using cell surface markers (see Supplementary Tables S2 and S3 for human and mouse conjugated antibodies, respectively). Living cells were incubated against antibodies resuspended into cold PBS free of Ca^2+^ and Mg^2+^, supplemented with 3% BSA and 2.5 mM EDTA on ice and were then fixed with PFA. Analysis was performed using a FACSCalibur Flow Cytometer (BD FACSCalibur cytometry System, San Jose, CA, USA) and data were analyzed with BD CellQuest Pro™ Software and Flowing Software 2.5.1 (Perttu Terho, Turku University, Turku, Finland).

### 4.7. Mesodermal Multipotency Analysis of Human OMC and MSC

The mesodermal multipotency of OMC was determined by analyzing their ability to undergo adipogenesis, chondrogenesis, and osteogenesis [51]. As such, OMC and MSC were cultured for 2 weeks into lineage-specific inductive media (for composition, see Supplementary Table S4). Adipogenic and osteogenic media were applied onto confluent OMC and MSC cultures. Media was changed each 72 h. For chondrogenesis, OMC and MSC spheroids were generated using suspension culture for 48 h into a Ultra Low Attachment Plate (3471, Corning Costar, Raleigh, NC, USA) as previously described [15]. Spheroids were transferred onto plastic adherent plates and cultured in the presence of chondrogenic medium for 10 days. Cells were finally fixed and processed for adipogenesis (Oil Red O), osteogenesis (Alkaline phosphatase), and chondrogenesis (Alcian blue) detection (see details see Supplementary Table S4).

### 4.8. RNA Isolation and Quantitative PCR Analysis of OMC and MSC

Total RNA was extracted from OMC and MSC cultures used for conditioned media production. Extraction was completed with RNeasy Mini Kit (Qiagen, Hilden, Germany) and RNA was reverse transcribed to cDNA with MMLV reverse transcriptase (Promega, Madison, WI, USA) as previously reported [13,15,26]. Quantitative real-time PCR was performed using SYBR Green and detected using an ABI Prism 7500 system (Applied Biosystems, Foster City, CA, USA). GAPDH was used to normalize gene expression. cDNA from MSC served as the calibrator sample and gene expression was set as 1. All primer sequences were from Primerbank (Supplementary Table S5).

### 4.9. Isolation of Mouse Lymphocytes

Lymphocytes were isolated from lymph nodes (LN) of adult C57BL/6J mice (H2b haplotype). Cervical, axillary, brachial, inguinal, lumbar, renal, pancreatic, and mesenteric lymph nodes were collected and gently disrupted into cold PBS + BSA between frosted margins of glass slides. Cells were then filtered through a 40 µm cell strainer, centrifuged, and incubated into red blood cells lysis buffer (Sigma-Aldrich). Freshly harvested mouse LN lymphocytes were characterized by IF and FC. Additionally, LN lymphocytes from adult FVB/N mice (H2q haplotype) were also isolated to perform a two-way mixed lymphocyte reaction (MLR) with LN lymphocytes from C57BL/6J mice. In some experiments, mouse lymphocyte MLR cultures were additionally stimulated with 25 µg/mL phytohemagglutinin (PHA-P; Sigma-Aldrich) to promote a strong proliferative response as indicated in initial lymphocytes cultures settings performed by our laboratory. For proliferation assays, mouse lymphocytes were stained for 8 min with 8 µm CFSE (formally known as 5-(and 6)-Carboxyfluorescein diacetate succinimidyl ester of CFDA SE) (Biotium, Fremont, CA, USA) diluted into a DMEM low glucose basal media (Gibco, 21885) and used after extensive washing. Additionally, peritoneal fluid cells (principally macrophages and B cells) were isolated from adult C57BL/6J mice and used to test the immunosuppressive activity of OMC-S and MSC-S.

### 4.10. Cytokine Array

A human Th1/Th2/Th17 cytokines antibody array kit (AB169809; Abcam, Cambridge, UK) was used to compare and analyze the secretion profile of Th1/Th2/Th17-related cytokines by OMC and MSC. To this end, membranes were either incubated into fresh media, or OMC-S and MSC-S (3 independent batches) and processed following the instructions of the manufacturer (see Protocol Booklet for Array Map). Chemiluminescence signal was captured with a ChemicDoc^TM^ MP Imaging System (Bio-Rad, Hercules, CA, USA) and quantified with the Image Lab 6.0.1 software (Bio-Rad). Exposure time was set using positive control spots in fresh media-incubated membrane (see Array Map). Cytokine signals were measured into a fixed circle area drawn around the spots. Basal signals for cytokines produced into a membrane incubated in fresh media was also calculated. The mean background signal for each PVDF membrane was also calculated into 5 areas lacking spot antibodies. Specific cytokine signal produced by OMC-S (*n* = 3) and MSC-S (*n* = 3) were finally calculated after subtracting non-specific basal and background signals. A grid (Figure 2) was drawn and added to the membrane’s pictures to delimitate in a single rectangle each cytokine (detected in duplicated dots).

### 4.11. M1/M2 Macrophage Polarization Study

The RAW 264.7 macrophage cell line was used as a well-defined cellular model to study M1/M2 macrophage polarization [52,53,54]. RAW 264.7 macrophage cell lines were expanded for two passages into fresh media (FM), which was the same media used for OMC and MSC expansion. RAW cells were finally equally distributed into 6-well plastic adherent plates (Nunc, Thermo Fisher Scientific, Rochester, NY, USA) and cultured for a period of 6 days into either FM or OMC-S or MSC-S. Cells were finally stained with cell surface markers (see Figure 3) and fixed with 4% paraformaldehyde prior to analysis by flow cytometry.

### 4.12. Human Peripheral Blood Mononuclear Cells Isolation

Peripheral blood was collected from healthy unrelated donors into heparinized tubes. Blood was diluted (1:1) with PBS, layered onto 10 mL Ficoll-Paque^TM^ PLUS (Amersham Biosciences, Buckinghamshire, UK) into a 50 mL conical tube, and centrifuged (400× *g*; 30 min) without break. PBMNCs were collected, resuspended into cold PBS-BSA-EDTA, and centrifuged (300× *g*; 5 min). PBMNCs were plated at a high density into tissue-culture-treated culture dishes in fresh media (FM) for 30 min to allow full adherence of myeloid cells. Free-floating lymphocytes were finally collected, centrifuged, and counted. These cells were termed PBMC-enriched lymphocytes in the study.

### 4.13. Quantification of Proliferation of Human Peripheral Blood Lymphocytes

The proliferation capacity of PBMC-enriched lymphocytes cultured into fresh media, OMC-S, or MSC-S was compared by using a two-way mix lymphocyte reaction (MLR) culture system supplemented with 25 µg/mL phytohemagglutinin (PHA) as described previously for mouse LN lymphocyte proliferation assays. Double polyclonal stimulation of PBMC-enriched lymphocytes was shown to promote a strong proliferation. Lymphocyte proliferation was measured by using the CFSE dilution method [55]. To this end, lymphocytes from two unrelated donors were stained for 8 min with 8 µm CFSE in basal media supplemented with 0.5% FBS and then washed twice in culture media before their use in experiments.

### 4.14. Measurements of Activated T and B Cells in Human Peripheral Blood Lymphocytes Cultures

Double polyclonal activation of T cells from peripheral blood lymphocytes was performed using the T cell TransAct^TM^, a polymeric nanomatrix structure with CD3 and CD28 antibodies (130-11-160, Miltenyi Biotec, Bergisch Gladbach, Germany) and addition of phytohemagglutinin (PHA-P; Sigma-Aldrich). To this end, the plastic non-adherent PBMNC fraction was collected and centrifuged. The pellet was then resuspended into 20 µL of fresh media containing 4 µL of T cell TransAct^TM^ for a period of 20 min to induce a strong activation of lymphocytes which were transferred to a culture dish containing fresh media and 25 µL/mL PHA to further improve T cell activation, agglutination, and proliferation. At day 3, activated lymphocytes, mainly in the form of aggregates, were collected, gently centrifuged, and incubated as described previously with T cell TransAct^TM^. Activated lymphocyte aggregates were transferred back to culture supplemented with fresh media for an additional 3 days of cultures. Lymphocytes were harvested after 6 days of expansion. Aggregates of activated T cells were then gently disaggregated and incubated for an additional 48 h in fresh media, OMC-S, or MSC-S. Cells were then analyzed for their co-expression levels of CD25 (activation marker) together with CD4 or CD19.

### 4.15. Culture Conditions of Mouse and Human Lymphocytes

Experiments with mouse or human lymphocytes were performed in P-24, P-48, or P-96 multiwell plates, being respectively seeded with a number of 8 × 10^6^, 4.10^6^, and 1 × 10^6^ of cells and a volume of culture media of 1 mL, 500 µL, or 200 µL.

### 4.16. Quantification of Lymphocytes Agglutination

The lymphosuppressive activity of OMC-derived secretomes was quantified by their ability to inhibit phytohemagglutinin-induced agglutination of mouse LN lymphocytes in 48 h culture experiments. Lymphocyte agglutination was initially induced by adding 25 µg/mL phytohemagglutinin (PHA) (Sigma-Aldrich; L1668). The capacity of diverse inhibitors to counteract the lymphosuppression activity of OMC was tested and inhibitors used were as following: TGF-β RI Kinase Inhibitor VI, SB431542, (Ref: 616461); NG-Methyl-L-arginine acetate salt or L-NMMA (Ref: M7033), L-Arginine (A5006), all from Sigma-Aldrich. Monoclonal antibody against TGF-beta I, II, III isoforms (Azide and BSA Free) was purchased from Novus Biological (Clone: 1D11.16.8; Ref: NBP2-47736). Cellular aggregation in the form of small to large spheroids was quantified with ImageJ (National Institutes of Health). Calculation of aggregates surface (µm^2^) was automated by converting images to binary images and processed to find hedges, prior to quantifying the mean particle size.

### 4.17. Statistical Analysis

Results are expressed as mean ± s.e.m (bar graphs). Statistical analyses were completed with GraphPad Prism software (GraphPad Software, La Jolla, CA, USA). Statistical differences were estimated by ANOVA or Student’s *t* test, whichever was appropriate.

## Data Availability

Data are available from the corresponding author on request.

## Supplementary Materials

The following supporting information can be downloaded at: https://www.mdpi.com/article/10.3390/ijms23115924/s1.

## References

[1] Mutsaers S.E. (2004). The mesothelial cell. Int. J. Biochem. Cell Biol..

[2] Mutsaers S.E., Wilkosz S. (2007). Structure and function of mesothelial cells. Cancer Treat. Res..

[3] Mutsaers S.E., Prele C.M., Lansley S.M., Herrick S.E. (2007). The origin of regenerating mesothelium: A historical perspective. Int. J. Artif. Organs.

[4] Mutsaers S.E. (2002). Mesothelial cells: Their structure, function and role in serosal repair. Respirology.

[5] Kawanishi K. (2016). Mesothelial cell transplantation: History, challenges and future directions. Pleura Peritoneum.

[6] Colmont C.S., Raby A.C., Dioszeghy V., Lebouder E., Foster T.L., Jones S.A., Labeta M.O., Fielding C.A., Topley N. (2011). Human peritoneal mesothelial cells respond to bacterial ligands through a specific subset of Toll-like receptors. Nephrol Dial. Transplant..

[7] Servais A.B., Kienzle A., Valenzuela C.D., Ysasi A.B., Wagner W.L., Tsuda A., Ackermann M., Mentzer S.J. (2018). Structural Heteropolysaccharide Adhesion to the Glycocalyx of Visceral Mesothelium. Tissue Eng. Part A.

[8] Katz S., Zsiros V., Kiss A.L. (2019). Under inflammatory stimuli mesenteric mesothelial cells transdifferentiate into macrophages and produce pro-inflammatory cytokine IL-6. Inflamm. Res..

[9] Liu M., Silva-Sanchez A., Randall T.D., Meza-Perez S. (2021). Specialized immune responses in the peritoneal cavity and omentum. J. Leukoc. Biol..

[10] Ivanov S., Gallerand A., Gros M., Stunault M.I., Merlin J., Vaillant N., Yvan-Charvet L., Guinamard R.R. (2019). Mesothelial cell CSF1 sustains peritoneal macrophage proliferation. Eur. J. Immunol..

[11] Riera M., McCulloch P., Pazmany L., Jagoe T. (2006). Optimal method for isolation of human peritoneal mesothelial cells from clinical samples of omentum. J. Tissue Viability.

[12] Pronk A., Leguit P., Hoynck van Papendrecht A.A., Hagelen E., van Vroonhoven T.J., Verbrugh H.A. (1993). A cobblestone cell isolated from the human omentum: The mesothelial cell; isolation, identification, and growth characteristics. Vitr. Cell. Dev. Biol. J. Tissue Cult. Assoc..

[13] Lachaud C.C., Lopez-Beas J., Soria B., Hmadcha A. (2014). EGF-induced adipose tissue mesothelial cells undergo functional vascular smooth muscle differentiation. Cell Death Dis..

[14] Mutsaers S.E., Di Paolo N. (2007). Future directions in mesothelial transplantation research. Int. J. Artif. Organs.

[15] Lachaud C.C., Pezzolla D., Dominguez-Rodriguez A., Smani T., Soria B., Hmadcha A. (2013). Functional vascular smooth muscle-like cells derived from adult mouse uterine mesothelial cells. PLoS ONE.

[16] Lansley S.M., Searles R.G., Hoi A., Thomas C., Moneta H., Herrick S.E., Thompson P.J., Newman M., Sterrett G.F., Prele C.M. (2011). Mesothelial cell differentiation into osteoblast- and adipocyte-like cells. J. Cell. Mol. Med..

[17] Van Tuyn J., Atsma D.E., Winter E.M., van der Velde-van Dijke I., Pijnappels D.A., Bax N.A., Knaan-Shanzer S., Gittenberger-de Groot A.C., Poelmann R.E., van der Laarse A. (2007). Epicardial cells of human adults can undergo an epithelial-to-mesenchymal transition and obtain characteristics of smooth muscle cells in vitro. Stem Cells.

[18] Elmadbouh I., Chen Y., Louedec L., Silberman S., Pouzet B., Meilhac O., Michel J.B. (2005). Mesothelial cell transplantation in the infarct scar induces neovascularization and improves heart function. Cardiovasc. Res..

[19] Elmadbouh I., Michel J.B., Chachques J.C. (2007). Mesothelial cell transplantation in myocardial infarction. Int. J. Artif. Organs.

[20] Asano T., Takazawa R., Yamato M., Takagi R., Iimura Y., Masuda H., Kihara K., Okano T. (2006). Transplantation of an autologous mesothelial cell sheet prepared from tunica vaginalis prevents post-operative adhesions in a canine model. Tissue Eng..

[21] Bresson L., Leblanc E., Lemaire A.S., Okitsu T., Chai F. (2017). Autologous peritoneal grafts permit rapid reperitonealization and prevent postoperative abdominal adhesions in an experimental rat study. Surgery.

[22] Kawanishi K., Nitta K. (2015). Cell sheet-based tissue engineering for mesothelial cell injury. Contrib. Nephrol..

[23] Kawanishi K., Nitta K., Yamato M., Okano T. (2015). Therapeutic applications of mesothelial cell sheets. Ther. Apher. Dial..

[24] Verhagen H.J., Heijnen-Snyder G.J., Pronk A., Vroom T.M., van Vroonhoven T.J., Eikelboom B.C., Sixma J.J., de Groot P.G. (1996). Thrombomodulin activity on mesothelial cells: Perspectives for mesothelial cells as an alternative for endothelial cells for cell seeding on vascular grafts. Br. J. Haematol..

[25] Kobori L., Nemeth T., Nemes B., Dallos G., Sotonyi P., Fehervari I., Patonai A., Slooff M.J., Jaray J., De Jong K.P. (2003). Experimental vascular graft for liver transplantation. Acta Vet. Hung.

[26] Lachaud C.C., Soria F., Escacena N., Quesada-Hernandez E., Hmadcha A., Alio J., Soria B. (2014). Mesothelial cells: A cellular surrogate for tissue engineering of corneal endothelium. Invest. Ophthalmol Vis. Sci.

[27] Lachaud C.C., Rodriguez-Campins B., Hmadcha A., Soria B. (2015). Use of Mesothelial Cells and Biological Matrices for Tissue Engineering of Simple Epithelium Surrogates. Front. Bioeng. Biotechnol..

[28] Lin C.Y., Kift-Morgan A., Moser B., Topley N., Eberl M. (2013). Suppression of pro-inflammatory T-cell responses by human mesothelial cells. Nephrol. Dial. Transpl..

[29] Kitayama J., Emoto S., Yamaguchi H., Ishigami H., Yamashita H., Seto Y., Matsuzaki K., Watanabe T. (2014). CD90(+)CD45(−) intraperitoneal mesothelial-like cells inhibit T cell activation by production of arginase I. Cell Immunol..

[30] Geiger R., Rieckmann J.C., Wolf T., Basso C., Feng Y., Fuhrer T., Kogadeeva M., Picotti P., Meissner F., Mann M. (2016). L-Arginine Modulates T Cell Metabolism and Enhances Survival and Anti-tumor Activity. Cell.

[31] Kim S.H., Roszik J., Grimm E.A., Ekmekcioglu S. (2018). Impact of l-Arginine Metabolism on Immune Response and Anticancer Immunotherapy. Front. Oncol..

[32] Eleuteri S., Fierabracci A. (2019). Insights into the Secretome of Mesenchymal Stem Cells and Its Potential Applications. Int. J. Mol. Sci..

[33] Herrick S.E., Mutsaers S.E. (2007). The potential of mesothelial cells in tissue engineering and regenerative medicine applications. Int. J. Artif. Organs.

[34] Hunter C.A., Jones S.A. (2015). IL-6 as a keystone cytokine in health and disease. Nat. Immunol..

[35] Mercalli A., Calavita I., Dugnani E., Citro A., Cantarelli E., Nano R., Melzi R., Maffi P., Secchi A., Sordi V. (2013). Rapamycin unbalances the polarization of human macrophages to M1. Immunology.

[36] Markov A., Thangavelu L., Aravindhan S., Zekiy A.O., Jarahian M., Chartrand M.S., Pathak Y., Marofi F., Shamlou S., Hassanzadeh A. (2021). Mesenchymal stem/stromal cells as a valuable source for the treatment of immune-mediated disorders. Stem Cell Res. Ther..

[37] Philipp D., Suhr L., Wahlers T., Choi Y.H., Paunel-Gorgulu A. (2018). Preconditioning of bone marrow-derived mesenchymal stem cells highly strengthens their potential to promote IL-6-dependent M2b polarization. Stem Cell Res. Ther..

[38] Jostock T., Mullberg J., Ozbek S., Atreya R., Blinn G., Voltz N., Fischer M., Neurath M.F., Rose-John S. (2001). Soluble gp130 is the natural inhibitor of soluble interleukin-6 receptor transsignaling responses. Eur J. Biochem..

[39] Ray A., Dittel B.N. (2010). Isolation of mouse peritoneal cavity cells. J. Vis. Exp..

[40] Yoshimoto M. (2020). The ontogeny of murine B-1a cells. Int. J. Hematol..

[41] Zhang F., Wang H., Wang X., Jiang G., Liu H., Zhang G., Wang H., Fang R., Bu X., Cai S. (2016). TGF-beta induces M2-like macrophage polarization via SNAIL-mediated suppression of a pro-inflammatory phenotype. Oncotarget.

[42] Espagnolle N., Balguerie A., Arnaud E., Sensebe L., Varin A. (2017). CD54-Mediated Interaction with Pro-inflammatory Macrophages Increases the Immunosuppressive Function of Human Mesenchymal Stromal Cells. Stem Cell Rep..

[43] Schutyser E., Struyf S., Van Damme J. (2003). The CC chemokine CCL20 and its receptor CCR6. Cytokine Growth Factor Rev..

[44] Sato K., Ozaki K., Oh I., Meguro A., Hatanaka K., Nagai T., Muroi K., Ozawa K. (2007). Nitric oxide plays a critical role in suppression of T-cell proliferation by mesenchymal stem cells. Blood.

[45] Valenti L.M., Mathieu J., Chancerelle Y., De Sousa M., Levacher M., Dinh-Xuan A.T., Florentin I. (2005). High levels of endogenous nitric oxide produced after burn injury in rats arrest activated T lymphocytes in the first G1 phase of the cell cycle and then induce their apoptosis. Exp. Cell Res..

[46] Valenti L., Mathieu J., Chancerelle Y., Levacher M., Chanaud B., De Sousa M., Strzalko S., Dinh-Xuan A.T., Giroud J.P., Florentin I. (2003). Nitric oxide inhibits spleen cell proliferative response after burn injury by inducing cytostasis, apoptosis, and necrosis of activated T lymphocytes: Role of the guanylate cyclase. Cell. Immunol..

[47] Aktan F. (2004). iNOS-mediated nitric oxide production and its regulation. Life Sci..

[48] Choi K.S., Song E.K., Yim C.Y. (2008). Cytokines secreted by IL-2-activated lymphocytes induce endogenous nitric oxide synthesis and apoptosis in macrophages. J. Leukoc. Biol..

[49] Lu L., Bonham C.A., Chambers F.G., Watkins S.C., Hoffman R.A., Simmons R.L., Thomson A.W. (1996). Induction of nitric oxide synthase in mouse dendritic cells by IFN-gamma, endotoxin, and interaction with allogeneic T cells: Nitric oxide production is associated with dendritic cell apoptosis. J. Immunol..

[50] Connell N.D., Rheinwald J.G. (1983). Regulation of the cytoskeleton in mesothelial cells: Reversible loss of keratin and increase in vimentin during rapid growth in culture. Cell.

[51] Crisan M., Yap S., Casteilla L., Chen C.W., Corselli M., Park T.S., Andriolo G., Sun B., Zheng B., Zhang L. (2008). A perivascular origin for mesenchymal stem cells in multiple human organs. Cell Stem Cell.

[52] Lv R., Bao Q., Li Y. (2017). Regulation of M1type and M2type macrophage polarization in RAW264.7 cells by Galectin9. Mol. Med. Rep..

[53] Ye Y., Xu Y., Lai Y., He W., Li Y., Wang R., Luo X., Chen R., Chen T. (2018). Long non-coding RNA cox-2 prevents immune evasion and metastasis of hepatocellular carcinoma by altering M1/M2 macrophage polarization. J. Cell. Biochem..

[54] Wang Z., Zhang X., Zhu L., Yang X., He F., Wang T., Bao T., Lu H., Wang H., Yang S. (2020). Inulin alleviates inflammation of alcoholic liver disease via SCFAs-inducing suppression of M1 and facilitation of M2 macrophages in mice. Int. Immunopharmacol..

[55] Di Carluccio A.R., Tresoldi E., So M., Mannering S.I. (2019). Quantification of Proliferating Human Antigen-specific CD4^+^ T Cells using Carboxyfluorescein Succinimidyl Ester. J. Vis. Exp..

