# Supplementary material for: Human Omental Mesothelial Cells Impart an Immunomodulatory Landscape Impeding B- and T-Cell Activation
Source: Int J Mol Sci. 2022 May 25;23(11):5924. doi: 10.3390/ijms23115924 (PMC9180401; doi:10.3390/ijms23115924)
Supplement: Supplementary file 1 [file ijms-23-05924-s001.zip › ijms-1661786-supplementary.pdf]

SUPLEMENTARY FIGURES

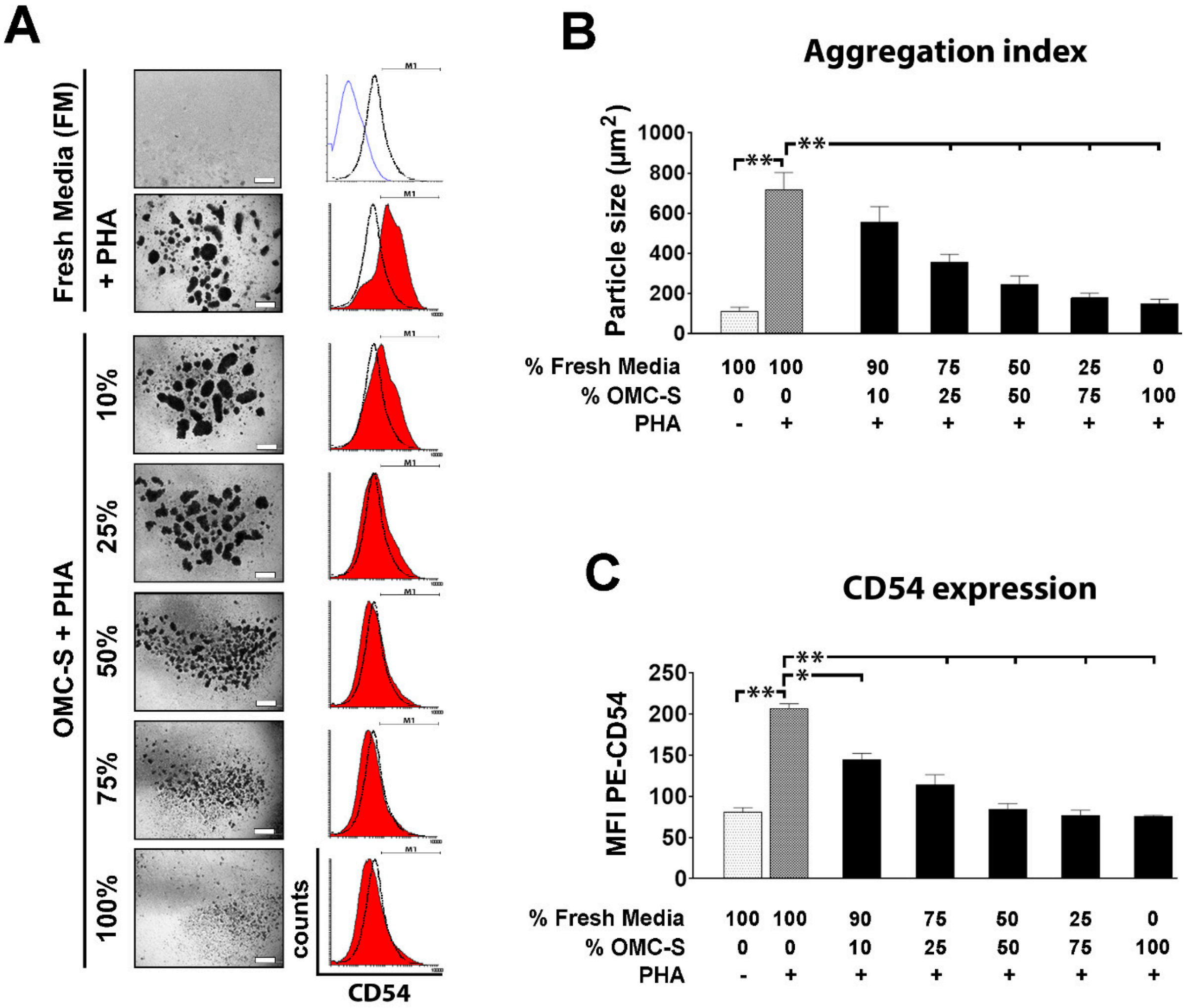

Figure S1: OMC-derived secretome inhibits PHA-induced agglutination and CD54 expression in mouse lymph nodes lymphocytes. (A) Left images show representative phase contrast pictures of mouse lymph nodes (LN) lymphocytes cultured for 48h either in fresh media (FM) supplemented or not with 25 μg/ml phytohemagglutinin (PHA). Note, the generation of large lymphocytes aggregates into FM supplemented with PHA. Alternatively, LN lymphocytes were cultured into increased proportions (10 to 100%) of OMC-conditioned media + PHA. Scale bar is 500 μm. Right images show corresponding flow cytometry histogram expression of intercellular adhesion molecule-1 (ICAM-1, CD54). In FM-PHA condition, blue line is for isotype expression. Dashed black line is for CD54 expression in FM-PHA condition, which is shown as reference in all PHA-stimulated conditions. Red filled histogram show CD54 expression. (B) Summary quantification of mean particle size (μm2) was performed as a direct measure of lymphocytes agglutination in response to experimental condition. (C) Summary quantification of CD54 mean fluorescent intensity (MFI) at 48h of experimental culture. (B-C) Experiment performed with (n=3) distinct OMC-S batches. Culture conditions are shown in the legend below graphs. Results are mean ± s.e.m. Statistical differences were calculated using ANOVA; * is for P≤0.05; ** is for P≤0.01.

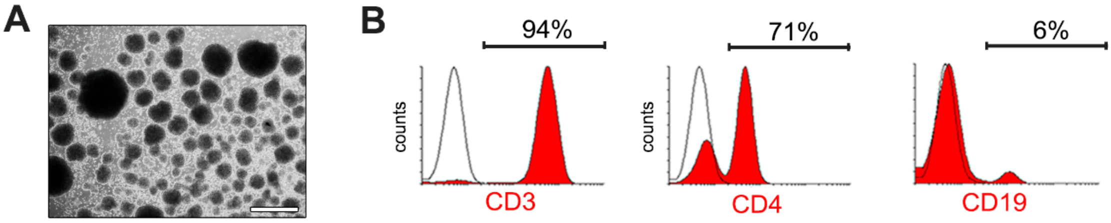

Figure S2: Characterization of human PBMC-enriched lymphocytes after combined polyclonal activation. (**A**) Shows representative aspect of PBMC-enriched lymphocytes subjected to a combined polyclonal CD3/CD28- and PHA-mediated activation (TCR and PHA) and cultured for 6 days. Lymphocytes grew as small to large floating spheroids, scale bar is 500µm. (**B**) Flow cytometric analysis of day 6 expansion cultures indicated that dissociated lymphocytes corresponded by overall majority to $CD3^{+}$ T cells, principally $CD4^{+}$ T cells, and also to a minor subpopulation of $CD19^{+}$ B cells.

SUPLEMENTARY TABLES

Table S1. **List of primary and secondary antibodies**

ANTIBODY	REF	DILUTION	COMPANY
Cytokeratin 18	MAB3234	1:200	Millipore
Wilm´s tumor protein 1 (WT1)	M3561	1:100	Dako
β-catenin	9581	1:200	Cell Signalling
Goat anti-mouse AF488	A11029	1:300	Life Technologies
Goat anti-rabbit AF488	A11034	1:300	Life Technologies

Table S2. **List of human conjugated antibodies.**

ANTIBODY	DILUTION	REF	COMPANY
FITC Mouse anti-human CD45	(1:50)	345808	BD
FITC Mouse anti-human CD31	(1:50)	555445	BD
FITC Mouse anti-human HLA-DR, DP, DQ	(1:50)	555558	BD
PE Mouse anti-human CD13	(1:50)	347406	BD
PE Mouse anti-human CD29	(1:50)	555443	BD
PE Mouse anti-human CD44	(1:50)	550989	BD
PE Mouse anti-human CD73	(1:50)	550257	BD
FITC Mouse Anti-Human CD90	(1:50)	555595	BD
PE Mouse anti-human CD105	(1:50)	560839	BD
PE Mouse anti-human CD166	(1:50)	559263	BD
PE Mouse anti-human CD54	(1:50)	560971	BD
PE Mouse anti-human CD3	(1:100)	555333	BD
FITC Mouse anti-human CD4	(1:100)	555346	BD
FITC Mouse anti-human CD8	(1:100)	555366	BD
FITC Mouse anti-human CD19	(1:100)	555412	BD
APC Mouse anti-human CD25	(1:100)	561399	BD
FITC Mouse IgG1, k		555748	BD
FITC Mouse IgG2a,k		555573	BD
FITC Mouse IgG2b,k		555742	BD

PE Mouse IgG1, k		555749	BD

Table S3. **List of mouse conjugated antibodies**

ANTIBODY	DILUTION	REF	COMPANY
FITC Rat anti-mouse CD11b	1:100	553310	BD
PE Rat anti-mouse CD54	1:100	553253	BD
PerCP-CyTM Hamster anti-mouse CD80	1:100	560526	BD
PE Rat anti-mouse CD86	1:100	561963	BD
PE Rat anti-mouse CD206	1:100	141705	Biolegend
BB515 Rat anti-mouse CD25	1:50	564424	BD
FITC Rat anti-mouse CD3	1:50	555274	BD
PE Rat anti-mouse CD4	1:50	553049	BD
PE Rat anti-mouse CD8	1:50	553032	BD
PE Rat anti-mouse CD19	1:100	557399	BD
PerCP-Cy™5.5 Hamster IgG2, κ	1:100	560562	BD
PE Rat IgG2a, k	1:100	553930	BD
BB515 Rat IgG1, λ	1:50	564417	BD
FITC Rat IgG2b, κ Isotype Control	1:100	553988	BD
PE Rat IgG2b, k	1:100	553989	BD

Table S4: **Composition of adipogenic, chondrogenic and osteogenic differentiation media.**

Adipogenic medium	Chondrogenic medium	Osteogenic medium
DMEM low glucose (Gibco)	DMEM low glucose (Gibco)	DMEM low glucose (Gibco)
10 % FBS (Hyclone)	1 % FBS (Hyclone)	10 % FBS (Hyclone)
1% P/S antibiotics (Gibco)	1% P/S antibiotics (Gibco)	1% P/S antibiotics (Gibco)
1X ITS (Gibco)	1X ITS (Gibco)	10 µM dexamethasone (Sigma; D4902)
1 µM dexamethasone (Sigma; D4902)	10 ng/ml rec. hum. TGF-ß1 (Peprotech; 100-21C)	50 µg/ml Ascorbic acid (Sigma; A7506)
250 µm 3-Isobutyl-1-methylxanthine (IBMX; Sigma; I7018)	50 ng/ml ascorbic acid (Sigma; A7506).	10 mM beta-glycerophosphate (Sigma; G9891)
250 µm indomethacin (Sigma; I7378)		
**DETECTION DIFFERENTIATION**		
Oil red (Sigma; O0625)	Alcian blue 8GX (Sigma; A5268)	SIGMAFAST BCIP/NBT substrate (Sigma; B5655)

Table S5: **List of primers used in the study**

Gene	Forward (5´--- 3´)	Reverse (5´--- 3´)
**IL-6**	ACTCACCTCTTCAGAACGAATTG	CCATCTTTGGAAGGTTCAGGTTG
**TGFβ**	CTAATGGTGGAAACCCACAACG	TATCGCCAGGAATTGTTGCTG
**IL-12a**	CCTTGCACTTCTGAAGAGATTGA	ACAGGGCCATCATAAAAGAGGT
**COX2**	TAAGTGCGATTGTACCCGGAC	TTTGTAGCCATAGTCAGCATTGT
**CXCL1**	AACCGAAGTCATAGCCACAC	GTTGGATTTGTCACTGTTCAGC
**IDO**	GCCAGCTTCGAGAAAGAGTTG	ATCCCAGAACTAGACGTGCAA
**ARG1**	TGGACAGACTAGGAATTGGCA	CCAGTCCGTCAACATCAAAACT
**IL-5**	TCTACTCATCGAACTCTGCTGA	CCCTTGCACAGTTTGACTCTC
**IL-1β**	ATGATGGCTTATTACAGTGGCAA	GTCGGAGATTCGTAGCTGGA
**iNOS**	TTCAGTATCACAACCTCAGCAAG	TGGACCTGCAAGTTAAAATCCC
**IL-2**	TCCTGTCTTGCATTGCACTAAG	CATCCTGGTGAGTTTGGGATTC
**IFNγ**	TCGGTAACTGACTTGAATGTCCA	TCGCTTCCCTGTTTTAGCTGC
**IL-15**	TTTCAGTGCAGGGCTTCCTAA	GGGTGAACATCACTTTCCGTAT
**IL-4**	ATGGGTCTCACCTCCCAACT	GATGTCTGTTACGGTCAACTCG
**TNFα**	CCTCTCTCTAATCAGCCCTCTG	GAGGACCTGGGAGTAGATGAG
**LGALS9**	GGGCGCAGACAAAAACCTC	GGAGTAGAGAACATCTGTCCAGG
**IL-10**	TCAAGGCGCATGTGAACTCC	GATGTCAAACTCACTCATGGCT
**GAPDH**	AGCCACATCGCTCAGACAC	GCCCAATACGACCAAATCC

Abbreviations: **IL-6**, interleukin 6; **TGFβ**, transforming growth factor-beta; **IL-12a**, interleukin 12 alpha; **Cox2**, cyclooxygenase-2; **CXCL1**, chemokine (C-X-C motif) ligand 1; **IDO**, Indoleamine-2,3-dioxygenase; **ARG1**, arginase I; **IL-5**, interleukin 5; **IL-1β**, interleukin 1 beta; **iNOS**, inducible nitric oxide synthase; **IL-2**, interleukin 2; **IFNγ**, Interferon gamma; **IL-15**, interleukin 15; **IL-4**, interleukin 4; **TNFα**, tumor necrosis factor alpha; **LGALS9**, galectin 9; **IL-10**, interleukin 10; **GAPDH**, Glyceraldehyde 3-phosphate dehydrogenase.
